# Global rise of potential health hazards caused by blue light-induced circadian disruption in modern aging societies

**DOI:** 10.1038/s41514-017-0010-2

**Published:** 2017-06-16

**Authors:** Megumi Hatori, Claude Gronfier, Russell N. Van Gelder, Paul S. Bernstein, Josep Carreras, Satchidananda Panda, Frederick Marks, David Sliney, Charles E. Hunt, Tsuyoshi Hirota, Toshiharu Furukawa, Kazuo Tsubota

**Affiliations:** 1 Department of Ophthalmology, Keio University School of Medicine, Shinjuku-ku, Tokyo Japan; 2 PRESTO, JST, Tokyo, Japan; 3 Univ Lyon, Universiteé Lyon 1, Inserm, Stem Cell and Brain Research Institute U1208, Bron, France; 4 Departments of Ophthalmology, Pathology, and Biological Structure, University of Washington School of Medicine, Seattle, USA; 5 Moran Eye Center, University of Utah, Salt Lake City, USA; 6 Catalonia Institute for Energy Research (IREC), Barcelona, Spain; 7 Regulatory Biology Laboratory, Salk Institute for Biological Studies, La Jolla, USA; 8 Academy of Neuroscience for Architecture, Salk Institute for Biological Studies, La Jolla, USA; 9 Consulting Medical Physicist, Fallston, USA; 10 Department of Electrical and Computer Engineering, University of California, Davis, USA; 11 Institute of Transformative Bio-Molecules, Nagoya University and PRESTO, JST, Nagoya, Japan; 12 Member of the House of Councilors, Japan. Keio University School of Medicine and School of Law, Tokyo, Japan

## Abstract

Mammals receive light information through the eyes, which perform two major functions: image forming vision to see objects and non-image forming adaptation of physiology and behavior to light. Cone and rod photoreceptors form images and send the information via retinal ganglion cells to the brain for image reconstruction. In contrast, nonimage-forming photoresponses vary widely from adjustment of pupil diameter to adaptation of the circadian clock. nonimage-forming responses are mediated by retinal ganglion cells expressing the photopigment melanopsin. Melanopsin-expressing cells constitute 1–2% of retinal ganglion cells in the adult mammalian retina, are intrinsically photosensitive, and integrate photic information from rods and cones to control nonimage-forming adaptation. Action spectra of ipRGCs and of melanopsin photopigment peak around 480 nm blue light. Understanding melanopsin function lets us recognize considerable physiological effects of blue light, which is increasingly important in our modern society that uses light-emitting diode. Misalignment of circadian rhythmicity is observed in numerous conditions, including aging, and is thought to be involved in the development of age-related disorders, such as depression, diabetes, hypertension, obesity, and cancer. The appropriate regulation of circadian rhythmicity by proper lighting is therefore essential. This perspective introduces the potential risks of excessive blue light for human health through circadian rhythm disruption and sleep deprivation. Knowing the positive and negative aspects, this study claims the importance of being exposed to light at optimal times and intensities during the day, based on the concept of the circadian clock, ultimately to improve quality of life to have a healthy and longer life.

## The circadian clock

Humans exhibit circadian rhythms in physiology, metabolism, and behavior that are essential to maintain organismal homeostasis in synchrony for 24 h a day. Chronic impairment of the circadian system has been shown to compromise health: disruptions of the circadian rhythms in shift workers are known risk factors for psychiatric disorders, gastrointestinal alterations, sleep and cognitive impairments, and breast cancer.^1, 2^ Since these non-infectious chronic diseases are also associated with aging, sustenance of circadian rhythmicity is a potential non-pharmacological approach to sustain healthy aging and hence reduce the global healthcare burden.

To improve clock function, it is necessary to understand the underlying mechanisms of circadian rhythmicity and the factors that perturb it. Almost all mammalian cells and tissues harbor cell-autonomous circadian clocks. Among them, the suprachiasmatic nucleus (SCN) in the hypothalamus serves as the master circadian clock controlling behavioral and physiological rhythms, and it is affected by environmental light exposure, while the peripheral clocks in tissues such as the liver are affected by food intake.^3^ Improvement in the feeding–fasting cycle that sustains a robust circadian clock in peripheral organs imparts health benefits. For example, wild-type mice fed a high-fat diet only during normal waking hours by restricting the time of feeding showed a larger amplitude of expression of circadian clock genes and staved off obesity, metabolic dysfunction, and liver damage compared to mice fed *ad libitum*, suggesting that when animals eat matters, not just what they eat.^4, 5^ Since chronic disruption of metabolism has been linked to increased incidence of non-communicable chronic diseases of aging, behavioral intervention (feeding–fasting) holds untapped cost-effective potential to improve quality of life.

## Melanopsin and non-image forming (NIF) vision

Our adaptation to ambient light is achieved by two basic mechanisms: image forming (IF) for vision and non-image forming (NIF) adaptation of physiology and behavior to light. IF function is mediated by the eye, with its specialized optical structures that project an image to the retina. Cone and rod photoreceptors of the outer retina capture the image and send the information via retinal ganglion cells (RGCs) to the visual cortex in the brain for image reconstruction. NIF photoresponses vary widely from adjustment of pupil diameter and increased vigilance and memory to slower responses, such as adaptation of the circadian clock to the daily day–night cycle and seasonal reproductive behavior in many animal species.^6^ NIF responses have been found almost intact in experimental animals and blind human patients with complete degeneration of rod/cone photoreceptors.

The cells mediating NIF responses express a photopigment melanopsin, are intrinsically photosensitive, and constitute 1–2% of RGCs in the adult mammalian retina, and hence they are known as intrinsically photosensitive RGCs or ipRGCs. Action spectra of ipRGCs and of melanopsin photopigment peak around 480 nm in the blue portion of the visible spectrum and match peak sensitivities of circadian photoentraiment, pupillary light reflex, light suppression of melatonin hormone, and light exacerbation of migraine pain.^6^ The NIF responses are attenuated, not eliminated, in melanopsin-deficient mice, and are completely abolished in mice that lack both melanopsin and functional outer retina photoreceptors. Specific ablation of ipRGCs leads to the loss of NIF responses, leaving IF responses nearly intact,^7–9^ pointing to ipRGCs as the site of functional integration of the rod/cone and melanopsin phototransduction pathways. ipRGCs project to the SCN and several other regions, including the intergeniculate leaflet of the thalamus, which indirectly entrains the circadian clock; structures involved in emotion, memory, and cognition; and a small midbrain structure, the olivary pretectal nucleus, which is the center controlling pupil constriction.^10^ Basic studies to understand the clock and melanopsin have been extensively done in not only rodent animal models but also in sighted and blind individuals, including aged subjects.^11–14^ Now we come to the novel concept that mammalian eyes work not only as an image capturing camera but also as a light sensor of the clock and NIF functions.

## Blue light and light-emitting diode (LED): Duality of blue light on human health

Understanding melanopsin function lets us recognize considerable physiological effects of blue light, which is increasingly important in modern society. After the invention of the incandescent light bulb in the late 19th century, followed by halogen light bulbs and fluorescent lamps in the 20th century, the easy access to light, even at nighttime, changed the human lifestyle, productivity, education, and industries. One of the recent innovations in the 21st century for the benefit of mankind is the light-emitting diode (LED), as represented by the 2014 Nobel Prize in Physics for the invention of the blue LED. Advantages of LED lighting over halogen bulbs are, among others, greater efficiency, less heat generation, improved life-cycle assessment, and substantially longer lifetimes. It is an international mandate to replace incandescent lights with more energy-efficient sources. Many countries have initiated national projects to replace standard light bulbs with LEDs to save energy. At present, LEDs are used in the displays for most flat-panel televisions and computer monitors, smartphones, and tablet devices. The latter two are the most recent inventions, with a human history of less than 10 years. Currently, the dominant method for LED lamps uses a combination of blue LED at 460 nm and a broad spectrum yellow garnet phosphor to produce “white” light, resulting in a far higher fraction of short-wavelength (460 nm blue) light than that produced by an incandescent light.^15^ Unfortunately, only the blue-LED/yellow-garnet LED is economically practical at this time, and it is likely that significantly higher amounts of blue wavelengths in LED light will be commonplace in the near future. The more balanced LED lamps using multiple colors (red, green, blue, amber) are substantially more complex and prohibitively expensive for general use,^15, 16^ but the situation may change in the long term.

The unanticipated revolution of LED lighting equipment has expanded its effect beyond providing light for vision: it also delivers a much higher amount of blue light compared to other light sources (incandescent, halogen, fluorescent).^15^ The blue component of light is known to have merits on health when used at appropriate timing and intensity.^17^ As previously mentioned, it is necessary for the synchronization of the circadian clock and the activation of NIF functions during the day. Light exposure is also used as an effective therapeutic in the treatment of depression and circadian sleep disorders, which has been termed “phototherapy” or “bright light therapy.”^18^ While the blue component of daylight peaks at midday, the extensive use of LEDs will possibly expose us to relatively higher amounts of blue light throughout the day, particularly in the evening and at night. Thus, compared to other lighting technologies (such as halogen, tungsten, and fluorescent light), potential hazards of extensive and/or long-term exposure to artificial blue light on our health, cognition, and aging, especially for infants to juveniles, cannot be ruled out. Light is known to affect human chronobiology more than any drug on the market.^17^ Its effects depend on the timing, intensity, duration, and spectral content of light. Since computer, e-reader, and smartphone displays contain high amounts of blue light, they may constitute a risk factor for sleep and circadian disorders. Epidemiological studies show a negative association between using electronic devices at night and sleep. According to a recently published study, using a tablet (e-reader) at night just before sleeping while in bed induces circadian phase delay and melatonin suppression, alters sleep quality, and reduces cognitive performance in the subsequent morning.^19^ It is proposed that exposure to bright light during the day can counteract the negative effect of using a tablet on sleep.^20^ Besides chronobiological impacts, LED screens could be phototoxic to the eye. Blue light has the highest photon energy in the visible spectrum, and it readily penetrates through the cornea and the lens to reach the retina, potentially resulting in retinal damage, a phenomenon known to ophthalmologists as the “blue light hazard.” Evidence of phototoxic effects of LED displays are lacking but they cannot be ruled out, in particular in conditions where intensity and/or duration of exposure are significant. Interestingly, blocking blue light protects the retina from light damage in mice, a finding that may have implications for the prevention of age-related macular degeneration in humans.^21^

## To avoid risks of blue light

Humans evolved in a world with solar-derived light spectra and an absence of light during the night. Flame lamps and fires emit little blue light. The studies described above strongly suggest that light of non-natural spectrum or timing may have profound and unintended effects on many parameters central to human physiology. We believe it is important that our colleagues, politicians, scientists, manufacturers, and, of course, citizens realize the potential risks of excessive blue light at night on human health. Based on the concept of the circadian clock and non-visual functions, it is critical to consider optimal timing during the day and the intensity of seeing and/or being exposed to blue light.^22^ The French Health Agency (ANSES) and the Scientific Committee on Emerging and Newly Identified Health Risks (SCENIHR), both in 2012, concluded that, although too early to draw a conclusion about the long-term effects of exposure to blue-rich light on human health, there was a potential phototoxic risk on the retina and a potential negative impact on sleep and non-visual/circadian biology. The American Medical Association (AMA) issued a warning report in 2013 entitled “Light Pollution: Adverse Health Effects of Nighttime Lighting.”^23^ The AMA recognizes that “exposure to excessive light at night, including extended use of various electronic media, can disrupt sleep or exacerbate sleep disorders, especially in children and adolescents.” The AMA also states that “disruption of circadian rhythmicity and sleep from the indiscriminate use of electric light at night may well increase risk of many of the diseases of modern life, including not only certain cancers but also obesity, diabetes, and psychiatric disorders.”

We launched the First International Symposium of the Blue Light Society in 2013 in Tokyo and are collaborating with the Japanese Ministry of Education, Culture, Sports, Science, and Technology (MEXT) to take initiatives to increase public awareness about the appropriate use of light-emitting devices that are blue-rich and to protect the population, in particular the youngest, from the potentially harmful impact of LED screens. It is necessary to ensure an educational system so that schoolteachers and parents fully recognize the possible effect of smartphones, tablets, computers, and TVs on people’s health. It is also important that the building industry, including architects and lighting offices, be responsible for maximizing the availability of natural daylight during daytime hours, in balance with reduced glare and increased energy efficiency, by adapting the light sources, design, and control mechanisms used in lighting. Technological advancements now make it possible to provide adjustable controls for daylight harvesting and electrical lighting that interact with electrochromic “switchable” glazing for windows and skylights. Similarly, light layering and dimmable lighting may offer personalized control over ambient light to enhance alertness during the active period and protect sleep during rest time.

We need more studies on the potential long-term effects of extensive exposure to blue-rich LED lighting on the human body, since it is a relatively recent invention. Children and adolescents are at particular risk for long-term exposure, but adults, including the aged, should not be forgotten. Due to the unavoidable 24/7 access and exposure to blue-enriched light, we need to provide adequate awareness among people based on the available scientific evidence. We propose that blue-enriched light, in particular but not exclusively from blue-containing LEDs, is a potential and an avoidable risk factor for health disorders.
